# Too much time or not enough? An observational study of teacher wait time after questions in case-based seminars

**DOI:** 10.1186/s12909-024-05667-w

**Published:** 2024-06-25

**Authors:** Janina Häusler, Martin Gartmeier, Marc Georg Grünewald, Alexander Hapfelmeier, Theresa Pfurtscheller, Tina Seidel, Pascal Oliver Berberat

**Affiliations:** 1 School of Health and Medicine, Technical University of Munich, TUM Medical Education Center, Chair for Medical Education, Munich, Germany; 2 School of Health and Medicine, Institute of General Practice and Health Services Research, Technical University of Munich, Munich, Germany; 3 School of Health and Medicine, Institute of AI and Informatics in Medicine, Technical University of Munich, Munich, Germany; 4 School of Social Sciences and Technology, Technical University of Munich, Friedl Schöller Endowed Chair for Educational Psychology, Munich, Germany

**Keywords:** Teacher wait time, Case-based learning, Interactive teaching, Dialogic teaching, Video study

## Abstract

**Background:**

We define teacher wait time (TWT) as a pause between a teacher question and the following response given by a student. TWT is valuable because it gives students time to activate prior knowledge and reflect on possible answers to teacher questions. We seek to gain initial insights into the phenomenon of TWT in medical education and give commensurate recommendations to clinical teachers.

**Methods:**

We observed *n* = 719 teacher questions followed by wait time. These were video-recorded in 29 case-based seminars in undergraduate medical education in the areas of surgery and internal medicine. The seminars were taught by 19 different clinical teachers. The videos were coded with satisfactory reliability. Time-to-event data analysis was used to explore TWT overall and independently of question types.

**Results:**

In our sample of case-based seminars, about 10% of all teacher questions were followed by TWT. While the median duration of TWT was 4.41 s, we observed large variation between different teachers (median between 2.88 and 10.96 s). Based on our results, we recommend that clinical teachers wait for at least five, but not longer than 10–12 s after initial questions. For follow-up and reproduction questions, we recommend shorter wait times of 5–8 s.

**Conclusions:**

The present study provides insights into the frequency and duration of TWT and its dependence on prior questions in case-based seminars. Our results provide clinical teachers with guidance on how to use TWT as an easily accessible tool that gives students time to reflect on and respond to teacher questions.

**Supplementary Information:**

The online version contains supplementary material available at 10.1186/s12909-024-05667-w.

## Background

Posing questions to students is an essential technique that clinical teachers use across a broad range of instructional formats, from bedside teaching to problem- or case-based learning. Questioning is characterized as “the simplest of interactive lecturing techniques, but often the most valuable” [1, p. 138]. By asking questions, clinical teachers involve students and motivate them to participate actively [2, 3]. Moreover, questions are useful in probing students’ knowledge about biomedical subject matter and guiding them to apply this knowledge, for instance, to patient cases. However, asking questions in pedagogically meaningful ways is far from straightforward: Good teacher questions take students’ prior knowledge into account and make sense in the broader context of a lecture or seminar [4–6]. The focus of the present study is on a further relevant feature of good teacher questions, namely the amount of time clinical teachers wait for students to respond after having posed a question.

We argue that *teacher wait time* (henceforth TWT) is a pedagogically relevant factor in verbal questioning processes as it allows students to reflect on teacher questions, activate prior knowledge [7, 8] and hence influences whether a question is responded to at all [9]. So far, the phenomenon of TWT has rarely been systematically investigated in medical education [10, 11]. This is unfortunate since evidence from school-related settings shows that if teachers provide ample wait time after questions, this leads to more elaborate and more correct responses, fewer *“I don’t know”* responses, and higher student engagement in class [1]. In the following, we describe why TWT after questions is important and summarize existing (mainly school-related) research findings on TWT. We report on our empirical low-inference video study in case-based seminars and casesaddress several research questions related to TWT: Among other aspects, we focus on the amount and length of TWT actually provided by clinical teachers and the variability of TWT between different teachers. Most importantly, however, we analyze *how much wait time clinical teachers should provide* in order to achieve high student response to their questions.

### Relevance of TWT in discursive teaching

An array of classroom studies show that providing sufficient TWT has positive instructional effects in interaction-based education [7, 12–18]. The value of wait time can be explained from both a cognitive and an emotional perspective. Authors investigating the cognitive perspective argue that TWT affords students more time to mentally process questions [9, 12, 18, 19] and thus provide more elaborate and provide correct answers.

Questions posed in medical education often demand high-level thinking [20]. It is our contention that TWT allows medical students to process teacher questions and reflect on possible answers [10], which in turn leads to fewer teacher questions remaining unanswered [21–23]. TWT is therefore of great importance in medical education: As a field with very high standards of expertise and professionalism, students may be reluctant to share their knowledge in situations where they are unsure.

The latter consideration also relates to the emotional perspective on TWT [13, 20]: Students may experience increased stress during periods of silence after teacher questions due to the conflict between hesitation (giving a potentially wrong answer) and intention to contribute to class and help advance an ongoing discussion. From this perspective, longer TWT might lead to increased emotional arousal and, as a consequence, involvement in class [13]. Conversely, students might experience frustration and detachment from class if they do not get enough time to properly think about possible answers to questions [20]. This study summarizes the evidence on how much wait time teachers *usually* provide and on how much wait time they *should* provide.

### How long do teachers commonly wait after posing questions?

Researchers have found that primary and secondary school teachers often wait only about one second before nominating a student [12, 19, 24, 25]. For higher education settings, Duell and colleagues reported an average TWT of 2.25 s after teacher questions. They argue that this average duration of TWT is too brief for students to think properly about (especially complex) questions [15]. The average TWT in Barrett’s study in medical education was 1.75 s [10]. However, this study focused on teaching occurring during surgical procedures (“intraoperative teaching”) – a rather specific clinical instructional setting. Jones investigated school classes regarding how much time students need to think prior to answering teacher questions [26]. On average, they found that students needed 2.8 s for closed questions and 6.9 s for open questions. In our empirical study, we also raise the question whether different amounts of wait time are appropriate after different types of questions.

In this respect, a challenge for research is that post-question wait time is often not terminated by student responses but by the teachers themselves, who respond to their own questions, reformulate questions or provide further information. In our study, we will also provide evidence on what kinds of teacher activities can be observed in cases when teachers, not students, terminate wait time.

### How long should teachers ideally wait after a question?

Rowe recommends at least three seconds, while Tobin considers three to five seconds after teacher questions to be appropriate [10, 18]. Concluding from a study in science education, Small also suggests that clinical teachers should wait three to five seconds before nominating a student [17]. Recommending a minimum TWT of three seconds, Heinze and Erhard conducted research in German mathematics classes and found that 75% of all teacher questions had shorter wait times [8]. Gage and Berliner also recommend a TWT of three to four seconds for repetitive questions and up to 15 s for questions that require thinking and reflection [24]. However, we argue that many empirical studies and the recommendations derived from these do not connect the given advice to specifically defined outcomes, e.g., on the level of student response. In our empirical study, we seek to advance existing research by empirically relating specific amounts of TWT to student response probabilities.

Drawing on the cognitive perspective outlined above, we argue that the more complex a question is, the more time teachers should allow students to ponder and respond. This relationship has already been analyzed in some empirical studies [27, 28]. Based on this evidence and theorization, we investigate how long *clinical teachers should ideally wait* after having posed a question. In the following, we will focus on different types of clinical teacher questions.

### TWT after different types of clinical teacher questions

We hypothesize that more complex teacher questions are associated with higher cognitive demands for students – and hence should be followed by longer TWT [27, 28]. In our study, we focus on several characteristics of teacher questions which represent different levels of cognitive demands, i.e., initial vs. repeated / follow-up questions, reproductive vs. reasoning and closed vs. open questions. These characteristics are explained in detail in the following:

When posing an *initial question*, a clinical teacher does not refer directly to a previously posed question. In contrast, *repeated questions* are rephrased versions of initial questions, e.g., when no student responds to an initial teacher question and the teacher poses a re-phrased version of the same question (often using simpler words). *Follow-up questions*, however, build on one or more previous student responses and/or previously posed, initial teacher questions. By posing follow-up questions, clinical teachers explore specific clinical aspects of a patient case in more depth. Here is an example of initial and follow-up questions (all examples in the following are taken or adapted from [5]):

#### Initial teacher question

“Which diagnostic procedures would you recommend at this point?”

#### Student response

“EKG, laboratory and sonography.”

#### Follow-Up teacher question

“Ok, and in which order would you conduct these procedures?”

Both repeated and follow-up questions build upon initial questions. This means students are already mentally engaged with the respective question topic and we expect to observe shorter TWTs after repeated and follow-up compared to initial questions.

Further, we investigate whether teachers should adapt wait time depending upon whether their questions have *closed* vs. *open* character. *Closed questions* have only one (or very few) correct response(s). For instance, in our study, one physician asked the students: *“What is the first thing you need to look at when examining a patients’ x-ray image?”* This is a typical closed question, as the only correct response is *“the patients’ name and the date the image was taken”* (in order to rule out that the image comes from another patient or is not the most recent one). In contrast, open questions afford more degrees of freedom. Questions such as *“What is your opinion on this clinical decision?* invite students to share their thoughts and verbalize their point of view. Due to the higher complexity of open questions, we expect longer wait times as compared to closed questions would be advisable.

Additionally, we focus on the cognitive level of teacher questions whereby we differentiate between *reproduction vs. elaboration (or reasoning) questions*. With *reproduction questions* (e.g., *“What is the MCH value, what does it tell you?”*), clinical teachers seek to elicit basic biomedical knowledge, e.g., from textbooks or previous courses. In contrast, through reasoning questions (e.g., *“Why would you recommend this therapy to this specific patient?“*), more complex cognitive processes are triggered, like describing cause-effect relationships or differentiating intended from unintended consequences of therapeutic measures. Responding to elaboration questions is more challenging compared to closed questions, as more information needs to be considered and combined as the basis of an appropriate answer. We hence hypothesize that, after elaboration questions, clinical teachers will need to provide more wait time in order to achieve similar response levels as compared to reproduction questions. To sum up, we address the following research questions:

RQ 1: How many teacher questions were followed by TWT and what was the median duration of TWT?

RQ 2: How much TWT should clinical teachers provide after initial vs. repeated/follow-up questions to achieve high student response rates?

RQ 3: Does the duration of TWT clinical teachers should provide vary depending upon different types of initial teacher questions they pose?

RQ 4: How does the duration of TWT vary between different clinical teachers?

RQ 5: How do clinical teachers themselves terminate teacher wait time?

## Methods

### Study design

We conducted a video study in a clinical undergraduate medical education environment. During the winter semester 2016/17, case-based seminars were video-recorded at TUM Rechts der Isar university hospital (henceforth TUM MRI). The cases discussed in the seminars were from the domains of surgery and internal medicine (each *n* = 16). To ensure the quality of the video recordings, trained staff followed a standardized recording procedure [29]. The study was open and non-participatory, i.e., all clinical teachers and students were informed prior to the video recordings about the means and purposes of the study. Moreover, the researchers who conducted the video recordings did not themselves participate in the seminars. Student participation in the study was voluntary. If students did not wish to participate, the seminar seating plan was re-organized so that the respective students were not videotaped.

### Ethics approval and data management

Approval for the present study was obtained in advance from the TUM MRI ethics committee (Application code 400/16 S). All methods were performed in accordance with the relevant guidelines and regulations. All videos and questionnaires were stored on the university’s internal drive in compliance with data protection guidelines. Only members of the study team had access to the stored data. All data will be stored for ten years, respecting standards of good scientific practice.

### Sample

**Fig. 1:**
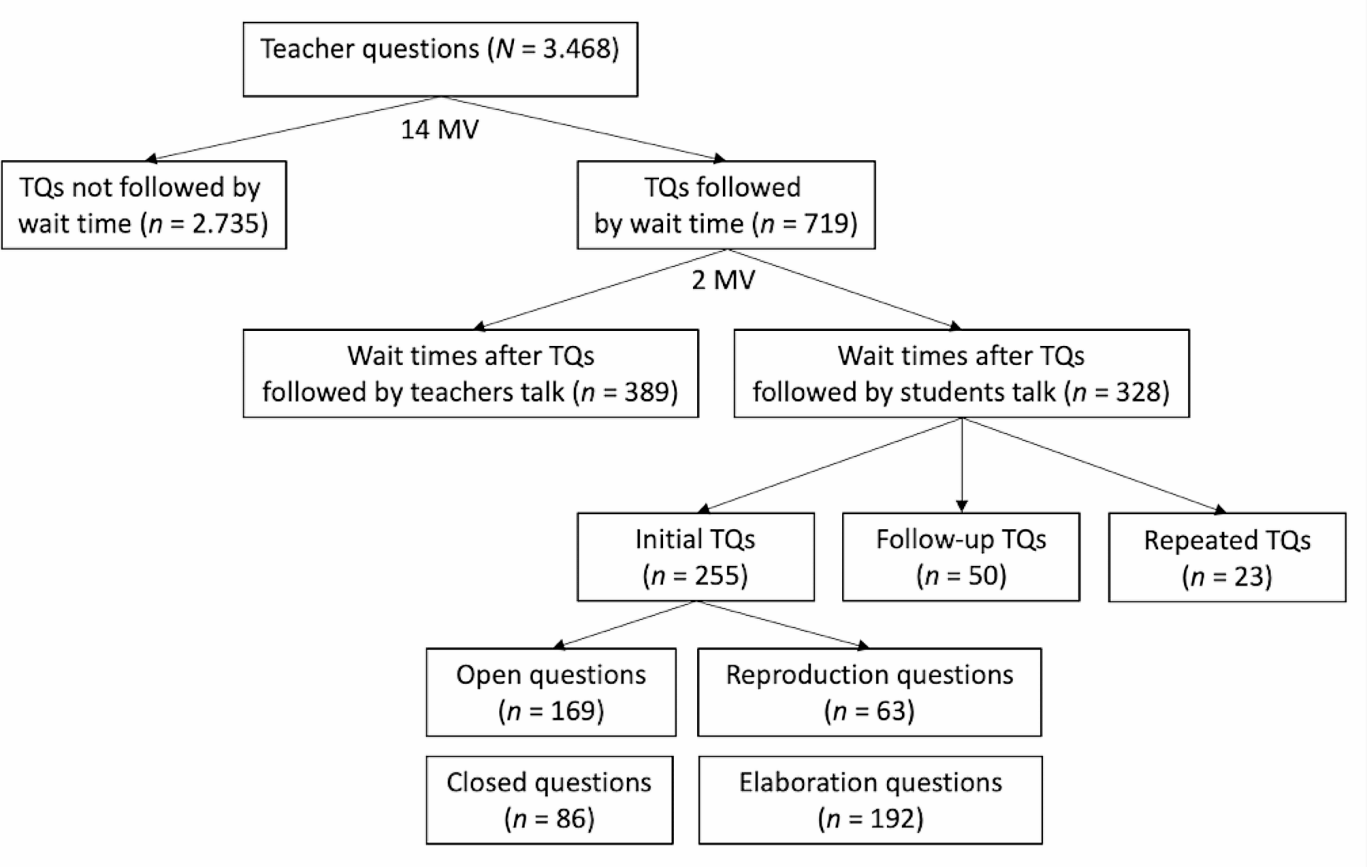
Sample overview *Note*: TQ = Teacher questions; MV = Missing values

The main focus of the present study is on content-related questions posed by clinical teachers in the context of case-based clinical seminars. In this respect, our sample comprises *N* = 3468 content related teacher questions and of these, *n* = 719 teacher questions followed by wait time (cf. Figure 1).

These questions were recorded during 32 clinical seminars, in which we filmed 21 different clinical teachers. Some teachers were video-recorded several times (two teachers were filmed five times and three were filmed twice). Due to audio problems, three videos could not be analyzed and were excluded. Therefore, only 29 videos from 19 clinical teachers were further analyzed; all information reported in the following relates to these 29 clinical seminars. Three of the 21 teachers were female, the rest were male. The teachers in our study were on average 39.5 years old (*Mdn* = 38, *SD* = 7.3, range = 31–57), and had been working as doctors for an average of 11.0 years (*Mdn* = 8.75, *SD* = 7.9, range = 4–30). Overall, 398 students were included in the study. On average, the participating students were 24.14 years old (*Mdn* = 23, *SD* = 2.95, range = 20–35), most of them were in the seventh (45.3%), eighth (35.5%) or ninth (15.3%) semester of their medical studies (*M* = 7.75, *SD* = 0.85, range = 6–11). On average, the seminars in our study were attended by 14 students (*min* = 7, *max* = 20). Teachers who were filmed several times taught different students and sometimes discussed different cases in the seminars, i.e., the conditions differed from seminar to *seminar*. Therefore, all videos of those clinical teachers were included.

### Video analysis

The video material was coded by four trained coders using the video analysis software Mangold Interact [30]. The video coding was performed in two steps: In the first step, the videos were segmented into speaking turns based upon *who was speaking*. Mangold Interact allows assigning codes directly to video material. The start/end time of each code was determined manually, with the accuracy determined by the frame rate of the underlying videos – this was 24 frames per second in our study. Possible segments or speaking turn codes were teacher / student male / student female / several or all students at the same time / pauses in dialogue / no one / external person. In the first round, we also coded the social form of classroom activities (such as plenary discourse or group work). In the present study, we focus only on phases of plenary discourse. In the second step of the coding process, each segment established in coding round one was coded for content (e.g., teacher statements: questions, explanations, classroom-management, etc.; student statements: questions, responses, other comments, etc.).

### Coding of TWT

In the second round of coding, we also coded TWT. We operationalize TWT as a pause after a question posed by a clinical teacher and a subsequent student contribution [12, 19].

In fluent, natural classroom conversations, slight pauses may occur between questions and responses, e.g., because a student is waiting to be called upon or is unsure if the teacher has actually finished talking. As such pauses cannot be considered TWT, we only coded pauses of *at least one second* as TWT [9]. Moreover, post-question wait time can also be terminated by the clinical teacher (e.g., by giving additional hints, rephrasing or answering the question). In such cases, students do not have the chance to respond to the question. In the present study, we report differentiated analyses for such cases. Further, the different types of teacher questions described above were coded using subcoding systems [5]. The overall interrater reliability showed a satisfactory Cohen’s kappa value of 0.65.

### Empirical analyses

We describe the distributions of our data using mean (*M*) and median values (*Mdn*) as well as minima (*Min*) and maxima (*Max*), interquartile range (*IQR*) and standard deviation (*SD*); this is because we found that many data distributions in our study were skewed, making mean values less meaningful. TWT was treated as time-to-event data as it measures the time after a teacher question until a subsequent verbal student contribution occurs. Kaplan-Meier estimates were used to describe the hypothetical probabilities (actuarial estimates) of TWT being terminated by a student, assuming that a teacher would not intervene earlier – i.e., censoring the event of a termination by a teacher [31]. Cox proportional hazards regression models were used to perform statistical hypothesis tests of group differences between question types at exploratory significance levels of 5%. Statistical analyses were conducted using SPSS version 25 (IBM Corp., Armonk, NY) and R 4.3.0 (The R Foundation for Statistical Computing, Vienna, Austria).

## Results

We report the results in the order of the posed research questions.

*RQ 1: How many teacher questions were followed by TWT and what was the median duration of TWT?*

Overall, we observed *n* = 719 teacher questions followed by a wait time (see Fig. 1). In such cases, the median wait time was *Mdn* = 3.40 s (*M* = 4.50 s; *IQR* = 2.22–5.68 s; *SD* = 3.49 s). As described above, the main focus of the present paper (and of the following analyses) are sequences of (initial) teacher question – wait time – student response. We observed this pattern in 328 cases and the median duration of wait time was *Mdn* = 3.18 s (*M* = 4.41 s, *IQR* = 2.32–6.26 s, *SD* = 3.74 s). Further, in 389 cases, instances of wait time were terminated by clinical teachers. In such cases, the median wait time edwas *Mdn* = 3.60 s (*M* = 4.59 s, *IQR* = 2.32–5.52 s, *SD* = 3.49 s).

*RQ 2: How much TWT should clinical teachers provide after initial vs. repeated/follow-up questions to achieve high student response rates?*

**Fig. 2:**
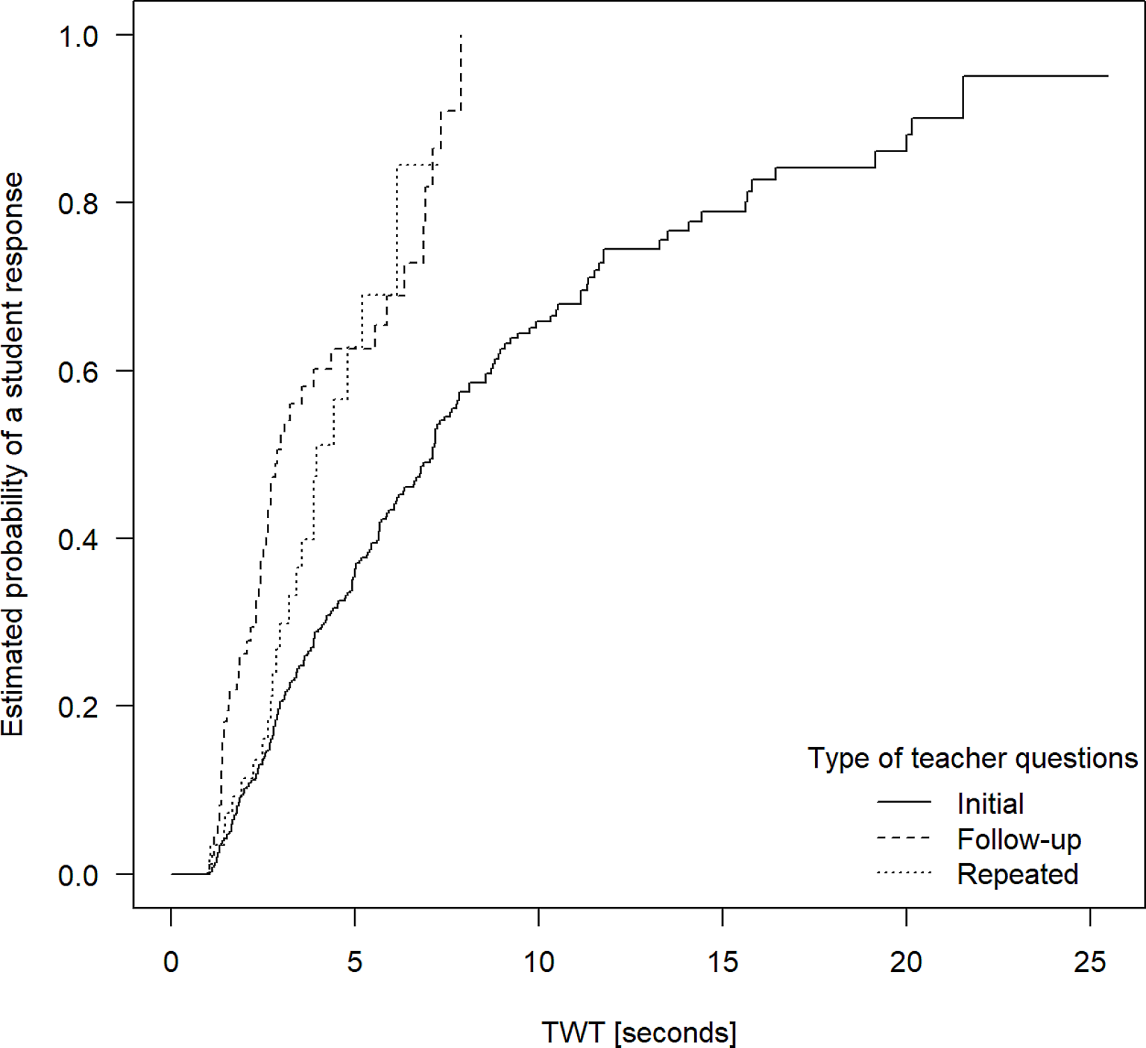
Probabilities of student responses with increasing TWT after initial, repeated and follow-up questions posed by clinical teachers

Figure 2,^1^ displays Kaplan-Meier curves showing the estimated probabilities of a wait time being terminated by a student, independent of TWT and type of question. As is apparent from Fig. 2, the chance of an initial teacher question being responded to after a TWT of five seconds was about 1/3 (36.4%, to be exact). After a TWT of ten seconds, this value increased further to about 2/3 (or 65.8%). Also, the initial teacher question response rate in Fig. 2 ascended constantly up to around the 12-second threshold (when about 75.4% of questions were responded to), after which the curve begins to flatten. This means allowing more than about 12 s of TWT still increased the chances of a student responding, but to a lower degree than previously.

Further, the Kaplan-Meier curves for repeated and follow-up questions indicate that for both these types of teacher questions, wait times of around five seconds were associated with an estimated probability of a student response of more than 60% (62.8% and 62.6%, respectively). Estimated student response rates of over 80% were reached after 7 s of TWT. The Cox regression model shows a statistically significant difference between initial and both, repeated and follow-up questions regarding the relationships described (both *p* < 0.001).

*RQ 3: Does the duration of TWT clinical teachers should provide vary depending upon different types of initial teacher questions they pose?*

**Fig. 3:**
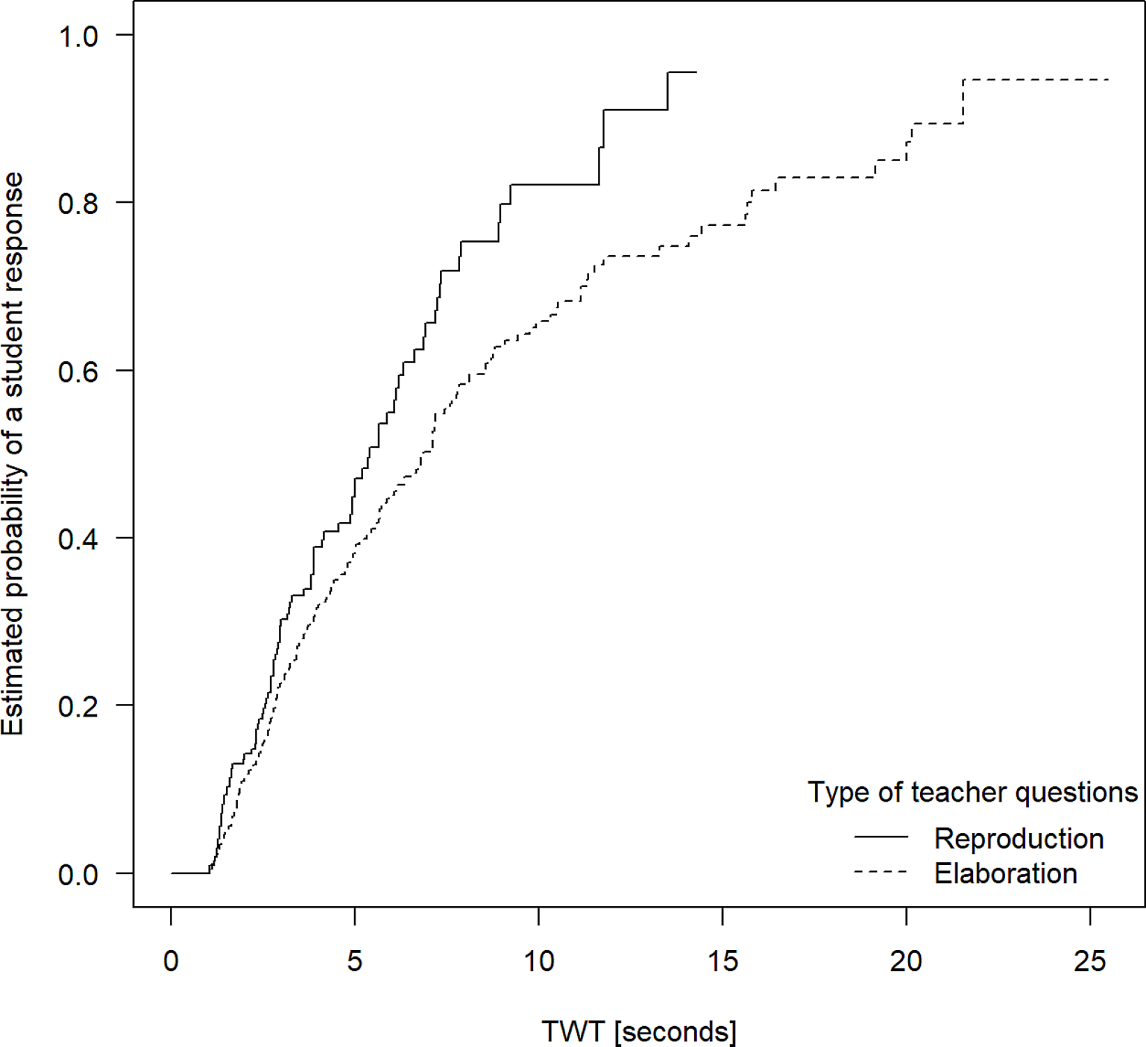
Kaplan-Meier curves showing estimated probabilities of reproduction and elaboration questions being responded to by students after different amounts of TWT

To analyze whether different recommendations regarding the appropriate amounts of wait time should be given for different types of initial teacher questions, we focus on reproduction vs. elaboration and closed vs. open questions. As Fig. 3 shows, elaboration-oriented teacher questions require clinical teachers to wait for longer time intervals until reaching the same student response probability value as compared to reproduction questions (*p* < 0.01). The chance of a reproduction question being responded to by a student after five seconds of TWT was 47.1%, whereas for elaboration questions it was only 38.5%.

**Fig. 4:**
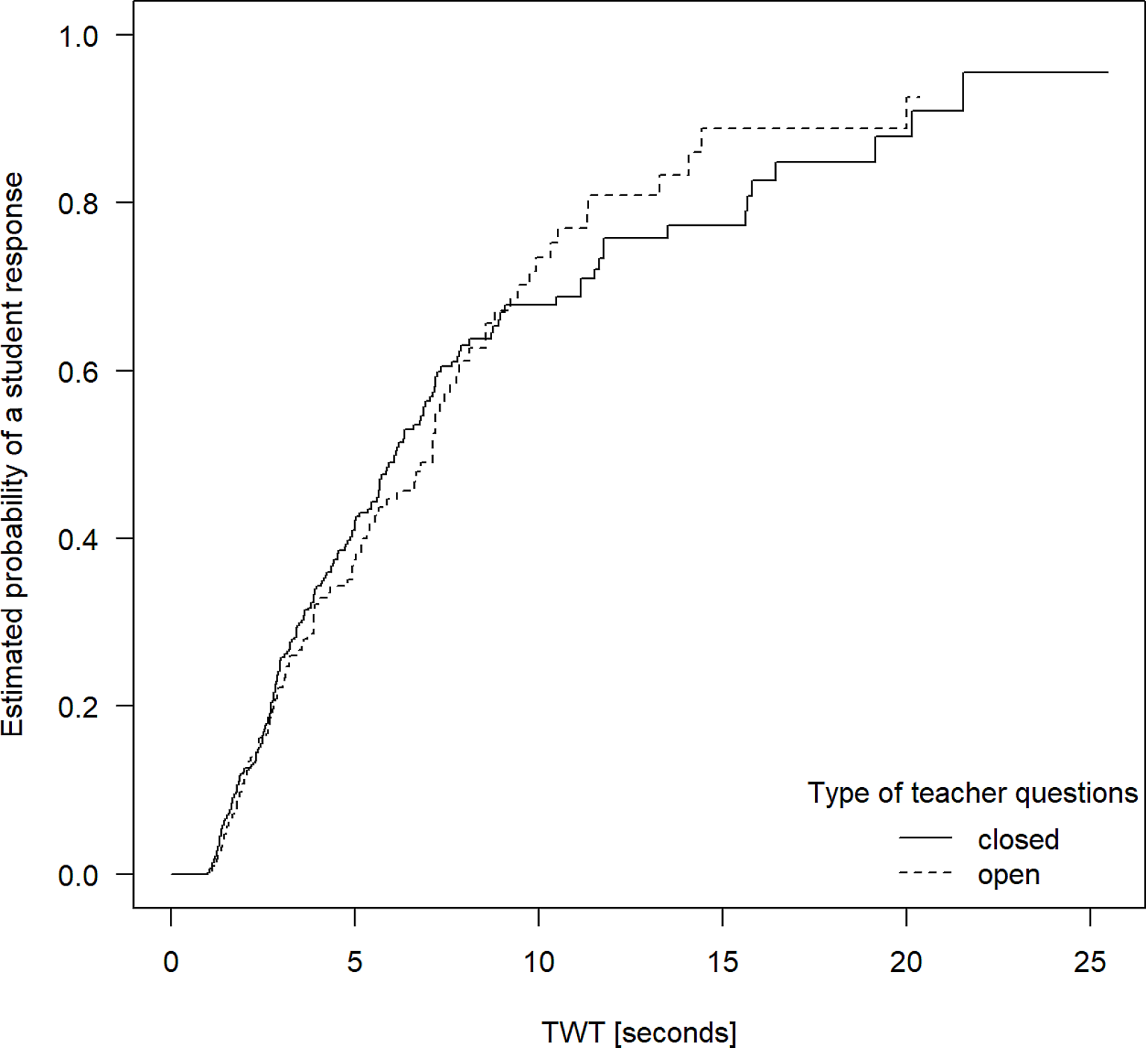
Probabilities of student responses with increasing TWT after closed vs. open questions posed by clinical teachers

Further, we compared closed and open-ended teacher questions. As Fig. 4 shows, the two curves are almost congruent, indicating no difference regarding the relation between TWT and student response probability regarding these question types (*p* = 0.86). Here, the probability of a teacher question being answered to after five seconds of wait time was 42.2% for closed and 37.5% for open questions – which is in the same range as for initial questions in general. After 10 s of wait time, the probability for obtaining a student response rose to about 67.8% for closed and 73.5% for open questions. As with initial teacher questions in general, a flattening of the increase of the curve after about 10 s is apparent.

*RQ 4: How does the duration of TWT vary between different clinical teachers?*

**Table 1:** Median duration of TWT for different teachers

Teacher	Median duration of TWT
1	2.88
2	3.16
3	3.60
4	4.28
5	4.80
6	4.80
7	4.96
8	4.98
9	5.76
10	6.16
11	6.32
12	6.92
13	7.04
14	7.20
15	7.44
16	7.52
17	7.87
18	10.96

*Note.* For one teacher, no median value can be reported as only one instance of wait time was observed

Table 1 shows substantial variation regarding the median amount of time different clinical teachers waited for a student response after having posed a question. Depending on which clinical teacher students were assigned to, the time to think about and answer teachers’ questions was therefore variable – from a median of just over two seconds to almost eleven seconds.

*RQ5: How do clinical teachers themselves terminate teacher wait time?*

**Fig. 5:**
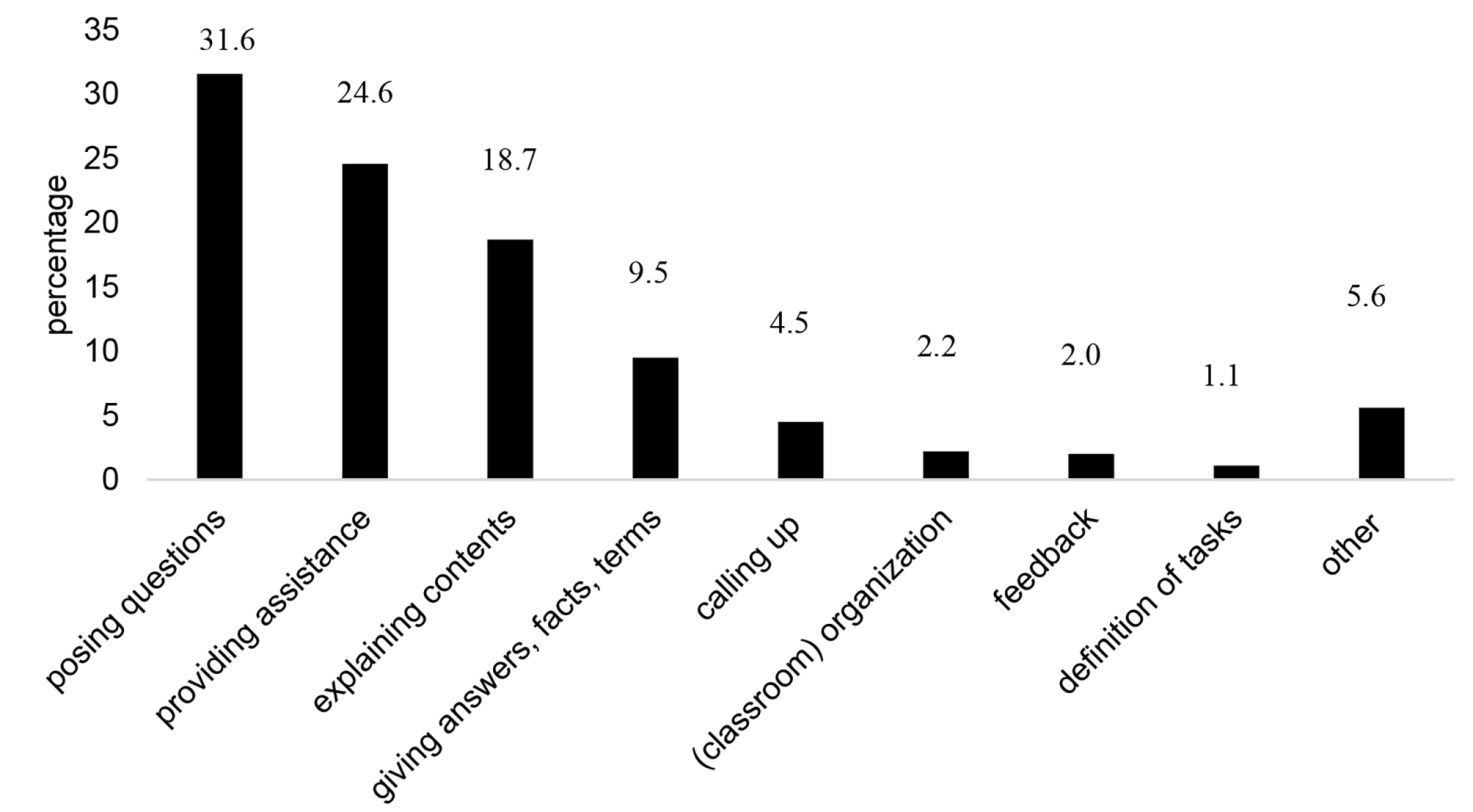
Teacher behaviors terminating instances of TWT

After instances of TWT that were not terminated by a student response, but by clinical teachers themselves, the most common teacher behaviors we observed were posing a further content-related question, providing further information to assist students, and further explaining the content of the medical case (see Fig. 5). The category “other” comprises activities such as giving examples and classroom management.

## Discussion

In this study, we examined teacher wait time as an aspect of instructional quality in case-based seminars in primary medical education. We defined TWT as the time interval between a clinical teacher question and the subsequent student response; by applying Kaplan-Meier estimators and plots, we were able to estimate probabilities of a student response after various durations of TWT.

Our results show that about 20% of teacher questions were followed by any form of teacher wait time, but only about 10% were followed by the sequence: wait time – student response. These findings are not in line with, for example, Li and Arshad’s study, in which 6.8% of all questions in chemistry classrooms were followed by a wait time [32]. The median TWT observed was 4.41 s in our study. This finding matches several authors’ [10, 17, 18, 24] suggestions that teachers should wait three to five seconds and exceeds the recommended wait time of three seconds advanced by Heinze and Erhard [8].

The further results of our study suggest specific recommendations for clinical teachers on how much time they should allow students to answer their questions: Based on our observations, after five seconds, responses to about 1/3 of initial questions can be expected and after 10 s, the value increases to about 2/3. However, after 12 s, longer wait times are associated with a smaller increase in the response rate. On this basis, we recommend that clinical teachers should always wait for at least five, but ideally for ten to twelve seconds, but not longer. Further, for follow-up questions, we found a response rate beyond 60% for a TWT of five seconds. Based on this finding, posing follow-up questions is effective in making the interaction in class more dynamic and stimulating students’ active participation. For follow-up questions, we recommend allowing five to eight seconds of wait time. Equally, clinical teachers should take into account the level of complexity of the questions they pose and provide more TWT for more complex (types of) questions, especially regarding reproduction / elaboration questions.

Further, we found large differences regarding the median duration of TWT different clinical teachers provided, ranging from 2.9 s up to more than 10 s. So, in some classrooms, teachers seem to provide students with ample time to reflect on the question, whereas in others, students have hardly any time to do so. This raises the question of whether some teachers in our study may have compromised students’ thinking processes by talking too much too soon after questions they posed.

Li and Arshad observe that TWT is most often followed by further teacher instructions or questions [32]. Our results confirm this: teachers in our study also tended to pose further questions (about 32%). Additionally, teachers in our study often provided students with further assistance and further explanations (25%) to help them respond to their questions.

Authors of prior studies on TWT conclude that teachers do not purposefully use TWT as an effective teaching method, as they could not find any connection between the observed wait times and the types of questions previously posed [7, 8]. Our results point towards a more diverse picture: TWT was closer to the suggested length of TWT as compared to prior studies [8, 18]. Moreover, we found different probabilities for responses after similarly long TWT in case of reproduction vs. elaboration questions, but not for closed vs. open questions. Previous studies underline the importance of differentiating TWT for different types of questions – but according to our results, this is the case for some, but not for all types of questions.

### Limitations and goals for future research

This video study provides empirical evidence on the use and relevance of TWT in the context of case-based seminars in medical education. However, we were only able to compare the results of our study with previous studies from other, mainly classroom contexts, but not with evidence from comparable instructional formats in medical education. We suggest further research in different pedagogical settings in the domain of medical education. Moreover, the fact that the present study was single center limits the generalizability of our results. It leaves open the question whether systematic differences regarding TWT exist in clinical teaching at different medical faculties, e.g., with respect to the average amount of TWT provided by clinical teachers or also as concerns the student response probabilities after TWT.

Regarding further insights into the value of providing ample TWT in interactive formats in medical education, an important question for future research is to what extent TWT can be associated with concrete outcomes on the level of learners, especially in terms of satisfaction, information retention, or motivation for self-directed learning. The design of our study did not allow us to address such questions.

Further, we cannot fully rule out that the clinical teachers in our study were influenced by the presence of our camera and modified their teaching, maybe towards a more interactive and student-centered approach. However, in a recent video-study on bedside teaching using a comparable methodological approach, no difference was observed between student evaluations of courses with vs. without a camera present [33]. Furthermore, the clinical teachers who took part in the study informally reported very little influence of the camera, either in the course of their bedside-teaching sessions or on their perception of the attending students’ behavior.

A further limitation of the present study is that we did not have a solid empirical or theoretical basis on which to differentiate between normal pauses in a fluent classroom interaction and TWT. We adopted the one-second threshold from existing classroom research as we were not able to identify more suitable evidence from higher or medical education. Future research should seek to gain more detailed, medical context specific insights. Other limitations on the methodological level concern the relative reliability of our codings and the fact that we filmed several teachers multiple times.

Finally, asking pedagogically fruitful questions that have a meaningful level of complexity, evoke relevant cognitive processes in learners and make sense in context of a specific lesson is a challenging task for clinical teachers [34]. Accordingly, TWT is only one of many aspects researchers should take into account when analyzing the instructional value of teacher questions [35]. In putting the focus on TWT, however, our point is not that just waiting a few seconds after posing a question makes the question itself more or less valuable from a pedagogical point of view. Rather, we argue that as medical education researchers and practitioners, paying attention to TWT is worthwhile – simply because the value of a question, no matter how meaningful in a specific situation, might be compromised, if students are not allowed enough time to mentally process the question.

### Conclusions for medical education and research

Following existing research and theorization [7, 12–18], the present study draws on the assumption that if clinical teachers provide wait time after posing a question, they create time for students to reflect on the question and increase the likelihood of eliciting an appropriate answer. Our results show that clinical teachers offered TWT to a larger extent and allowed longer TWT than teachers in numerous prior studies. This inspires new questions for further research. First, how does this difference relate to the contextual conditions in schools and higher, and especially, medical education? Our results show that the phenomenon of TWT is quite complex, so we recommend further studies on different pedagogical formats of medical education (as well as in other subject areas in higher education). Furthermore, it would be interesting to compare our results to other interactive formats of medical education (like bedside or simulation-based teaching). In this respect, video studies offer a very promising approach [10]. Second, as this is mandatory at the respective institution, all clinical teachers filmed in our study participated in a one-day workshop regarding university teaching. Some may even have participated voluntarily in further pedagogical training programs offered at our university. Therefore, in a future study, an interesting research question could target relationships between participation in teacher training and the extent of TWT provided in class.

In our study, we also found substantial heterogeneity regarding the duration of TWT between teachers. Clinical teachers should be sensitive to whether the students they instruct may need more or less time to reflect before sharing their thoughts with the group. Our findings suggest that there is room for improvement in how teachers use TWT in class. As consequence of this, the issue of TWT has been integrated into the teacher training curriculum for clinical teachers at our institution.

### Electronic supplementary material

Below is the link to the electronic supplementary material.

Supplementary Material 1

## Data Availability

The datasets used and analyzed in the current study are available from the corresponding author on reasonable request.

## Footnotes

1 Please see Table A1 in the appendix for the exact figures behind Figs. 1, 2 and 3.

## Abbreviations

TWT: Teacher wait time
