# Supplementary material for: Too much time or not enough? An observational study of teacher wait time after questions in case-based seminars
Source: BMC Med Educ. 2024 Jun 25;24:690. doi: 10.1186/s12909-024-05667-w (PMC11202393; doi:10.1186/s12909-024-05667-w)
Supplement: Supplementary file 1 — Supplementary Material 1 [file 12909_2024_5667_MOESM1_ESM.docx]

**Table A1**

Estimated probabilities of different types of teacher questions being responded to by students after varying amounts of TWT

| TWT [seconds] | Response probabilities [percent] | | | | | | |
| --- | --- | --- | --- | --- | --- | --- | --- |
|  | Initial questions  overall | Subtypes of initial questions | | | | Repeated questions | Follow-up questions |
|  |  | Closed  questions | Open questions | Reproduction  questions | Elaboration questions |  |  |
|  | Estimated student Response probability | | | | | | |
| 1 | 0.2 | 0.2 | 0.0 | 0.0 | 0.2 | 0 | 0 |
| 2 | 10.3 | 12.6 | 11.3 | 14.2 | 11.5 | 11.4 | 26.2 |
| 3 | 20.4 | 25.8 | 22.2 | 30.3 | 22.7 | 29.9 | 52.3 |
| 4 | 29.2 | 34.3 | 32.8 | 38.9 | 32.1 | 51.2 | 60.2 |
| 5 | 36.4 | 42.2 | 37.5 | 47.1 | 38.5 | 62.8 | 62.6 |
| 6 | 43.4 | 49.0 | 44.7 | 55.0 | 45.1 | 69 | 68.9 |
| 7 | 49.1 | 56.3 | 49.1 | 65.6 | 50.2 | 84.5 | 81.9 |
| 8 | 57.5 | 63.1 | 61.2 | 75.4 | 58.3 |  | 100.0 |
| 9 | 62.6 | 66.9 | 67.1 | 79.8 | 62.8 |  |  |
| 10 | 65.8 | 67.8 | 73.5 | 82.1 | 65.8 |  |  |
| 11 | 68.0 | 68.8 | 77.0 | 82.1 | 68.3 |  |  |
| 12 | 74.5 | 75.7 | 80.8 | 91.0 | 73.6 |  |  |
| 13 | 74.5 | 75.7 | 80.8 | 91.0 | 73.6 |  |  |
| 14 | 76.6 | 77.3 | 83.2 | 95.5 | 74.8 |  |  |
| 15 | 78.9 | 77.3 | 88.8 |  | 77.3 |  |  |
| 16 | 82.7 | 82.7 | 88.8 |  | 81.4 |  |  |
| 17 | 84.1 | 84.8 | 88.8 |  | 82.9 |  |  |
| 18 | 84.1 | 84.8 | 88.8 |  | 82.9 |  |  |
| 19 | 84.1 | 84.8 | 88.8 |  | 82.9 |  |  |
| 20 | 88.1 | 87.9 | 95.7 |  | 87.2 |  |  |
| 21 | 90.1 | 90.9 |  |  | 89.3 |  |  |
| 22 | 95.0 | 95.5 |  |  | 94.7 |  |  |
| 23 | 95.0 | 95.5 |  |  | 94.7 |  |  |
| 24 | 95.0 | 95.5 |  |  | 94.7 |  |  |
| 25 | 95.0 | 95.5 |  |  | 94.7 |  |  |
